# Occupational cold stress and rewarming alters skin temperature thresholds for manual dexterity decrements: An exploratory study

**DOI:** 10.14814/phy2.70342

**Published:** 2025-05-08

**Authors:** Christopher L. Chapman, Brandon M. Roberts, Erica A. Schafer, John W. Castellani, Karl E. Friedl, Adam W. Potter, David P. Looney

**Affiliations:** ^1^ United States Army Research Institute of Environmental Medicine (USARIEM) Natick Massachusetts USA; ^2^ Oak Ridge Institute for Science and Education Oak Ridge Tennessee USA; ^3^ Maximize Human Performance, LLC Framingham Massachusetts USA; ^4^ CoachMePlus Buffalo New York USA

**Keywords:** cold injury, environmental, fingers, hands, work‐rest

## Abstract

The skin temperature thresholds at which precipitous reductions in dexterity occur in cold environments, and whether they are altered by rewarming, are not well defined. In three environmental conditions (20°C, 10°C, and 0°C air temperatures), 14 healthy adults (three females; age: 24 ± 6 years) completed five dexterity tests (Placing Test) over ~130 min of various light‐to‐moderate physical activities to simulate occupational work demands while minimally dressed. Brief passive rewarming (10 min in ~22°C air temperature) and a final dexterity test upon reentry to the environment was then performed. Dexterity was evaluated as the absolute (seconds) or percent change from an individual's best baseline performance. Prior to rewarming, segmented regression revealed thresholds for greater dexterity loss during progressive cold strain occurred at skin temperatures of ~22.9°C (fingers), ~24.9°C (hand), and ~22.4°C (forearm) (all *p* ≤ 0.002). After rewarming, this threshold shifted upwards to ~25.7°C for the fingers (*p* ≤ 0.007). The hand skin temperature threshold after rewarming was ~27.1°C (for absolute changes, *p* < 0.001), but one was not identified with percent change (*p* = 0.074). A forearm skin temperature threshold was not identified following rewarming (*p* ≥ 0.058). These findings indicate that, in non‐hypothermic conditions, skin temperature thresholds for dexterity loss during prolonged occupational cold stress may be modified with rewarming.

## 1 INTRODUCTION

Occupational cold stress elicits an integrative physiological response in humans that heightens metabolic strain (Schafer et al., 2024), induces cutaneous vasoconstriction (Alba et al., 2019), and causes marked decrements in manual dexterity (Ray et al., 2019). Manual dexterity reflects the coordinated movements between the fingers and hands to grasp and manipulate objects. Importantly, manual dexterity is a fundamental element of many occupations and recreational activities (e.g., military personnel, first responders rendering medical aid, electrical utility line workers, heavy machinery operators, and mountaineering). Decrements in manual performance can increase the risk of adverse health and safety outcomes, potentially turning a survival situation into a critical one (Cheung, 2015), especially if involving accidental immersion in cold water (Cheung et al., 2003; Jones et al., 2023). The observation that cold exposure reduces manual dexterity has been well documented over the years (Castellani & Tipton, 2016; Havenith et al., 1995). However, countermeasures to effectively evade these manual dexterity decrements continue to be an important capability gap in military operations and occupational settings as, for example, glove thickness independently reduces dexterity (Havenith et al., 1995) and local heating devices require battery power that ceases to function in extreme cold (Sullivan‐Kwantes et al., 2021).

Impairments in manual dexterity during cold stress are related to a number of factors, including reductions in tactile sensitivity (Provins & Morton, 1960), joint mobility (Hunter et al., 1952), and neuromuscular function (Mallette et al., 2018; Racinais & Oksa, 2010). These factors are further modulated by the rate of cooling (Ray et al., 2019) and whether the cooling is occurring centrally or peripherally (Cheung & Sleivert, 2004; O'Brien et al., 2007). As such, body heat storage, which is the cumulative change in body heat content over time (Cramer & Jay, 2019), is predictive of finger temperature and manual dexterity during cold exposure (Brajkovic et al., 2001; Brajkovic & Ducharme, 2003). In general, manual dexterity decrements are observed at finger skin temperature thresholds between 12°C and 20°C (Ray et al., 2019), although 15°C is typically regarded as the finger temperature threshold for marked decrements in dexterity (Castellani & Tipton, 2016; TBMED‐508, 2005) and hand temperature thresholds are typically around 22°C (Kingma et al., 2023). These thresholds are likely dependent on the severity and duration of the cold exposure as well as whether individuals are exercising and wearing gloves. For example, in one study, thermal face protection (i.e., balaclava and goggles) attenuated reductions in finger and hand temperatures for up to 30 and 60 min, respectively, while standing during cold air exposure (−15°C with headwind of 3 m/s) (O'Brien et al., 2011). In another study, a 1‐h exposure to −1°C when participants were appropriately dressed did not result in a decrement in manual dexterity as hand temperatures were reduced to only ~21–22°C (Orysiak et al., 2024).

In many occupational settings and military operations, individuals may be required to perform prolonged light‐to‐moderate intermittent physical work (>1 h) in cold environments while experiencing progressive decrements in manual dexterity that threaten task performance and their health and safety. In these often remote environments, individuals may only be allowed or have access to brief respite from the cold in the form of a warming shelter without additional active heating modalities. Recently, it was found that maximal vasoconstriction in the fingers occurred at local skin temperatures between ~26°C and 28°C and that vasoconstrictor withdrawal occurred at similar local finger temperatures (~25°C–27°C) during gradual passive rewarming (rate of increased air temperature of +0.5°C per min) (Massey et al., 2018). These findings indicate that the local skin temperature threshold for cutaneous vasoconstrictor tone does not differ between cooling and rewarming. However, whether the local skin temperature thresholds indicative of greater manual dexterity decrements are modified after passive rewarming requires further study, especially in dynamic conditions of intermittent work as is commonly experienced during occupational cold stress.

Therefore, the purpose of the present study was to (a) identify thresholds in extremity skin temperatures (i.e., finger, hand, and forearm) for dexterity loss during light‐to‐moderate physical activity during progressive cold stress and brief rewarming and (b) determine diagnostic accuracy of finger, hand, and forearm skin temperatures, core temperature, and thermal sensation for discriminating a 5% and 10% loss of dexterity in this paradigm. We hypothesized that finger, hand, and forearm skin temperatures would exhibit statistical thresholds where the decrement in manual dexterity for a given reduction in extremity skin temperature becomes more pronounced.

## 2 METHODS

### 2.1 Ethical approval

This study was approved by the Institutional Review Board at the United States (US) Army Medical Research and Development Command (MRDC; Fort Detrick, MD, USA) (Approval number M‐11018). Investigators adhered to Department of Defense Instruction 3216.02 and 32 CFR 219 on the use of volunteers in research. All participants were briefed on the study and its potential risks prior to providing their written informed consent. This study conformed to the standards set by the latest version of the Declaration of Helsinki, with the exception of registration in a database.

### 2.2 Participants

Fourteen healthy adults (sex assigned at birth: three females and 11 males; age: 24 ± 6 years; height: 172 ± 8 cm; body mass: 72 ± 16 kg, body fat: 21.5 ± 8.2%, and body surface area: 1.82 ± 0.19 m^2^) participated in this study, including 10 active‐duty US Army Soldiers (three females) and four male civilians. Participants were screened by a physician prior to enrollment in the study. Participants were deemed eligible for the study with ages between 18 and 44 years and having a minimum physical activity of 2 days per week of aerobic or resistance exercise for at least 30 min. Exclusion criteria for the study were as follows: (1) musculoskeletal injuries or medical conditions that compromise the ability to exercise; (2) pregnancy; (3) claustrophobia or difficulty breathing into a mouthpiece; (4) history of frostbite, cold‐induced asthma, Raynaud's syndrome, and/or nonfreezing cold injuries; (5) contraindications to the telemetry pill used to measure core temperature, including history of serious gastrointestinal disease, previous serious gastrointestinal surgery, possible nuclear magnetic resonance imaging (MRI) scan within 2 weeks, or difficulty swallowing large pills; (6) current use of serotonergic, diuretic, myopathic, or nephrotoxic medication, furosemide, and anticoagulant medication.

### 2.3 Measurements and instrumentation

Height was measured using a stadiometer (Model 213, Seca, Hamburg, Germany) on visit 1. Nude body mass was measured with a standard calibrated scale (Model 876, Seca, Hamburg, Germany) on every visit. On visit 2, prior to instrumentation, three‐dimensional body scans were used to measure body surface area (SS20 Booth Scanner, Size Stream LLC; Cary, NC) and body composition was assessed by dual‐energy X‐ray absorptiometry (DPX‐IQ, Lunar Corporation, Madison, WI). Core temperature was measured using a telemetry pill (eCelsius Performance capsule, BodyCAP, Herouville‐Saint‐Clair, France) sampling once every 15 s that was self‐inserted as a suppository approximately 7–8 cm into the rectum (*n* = 13) or was swallowed the night before the study visit (*n* = 1). Extremity skin temperature was measured via wireless temperature sensors (Thermochron iButton model DS1922L, Maxim Integrated, San Jose, CA) with a resolution of 0.0625°C sampling every 60 s affixed to the skin at the dorsal aspect of the proximal phalanx of the fourth finger, dorsal aspect of hand, and the ventral aspect of forearm. Skin temperature was measured on the hand and forearm because these are more directly translatable sites that may be measured in real‐time field environments, such as military training, where bulky sensors are an impediment to critical tasks. Heart rate was measured using the eq02+ Lifemonitor (Equivital, Hidalgo Ltd., Cambridge, UK) sampling at 256 Hz and proper fitting was ensured per manufacturer's instructions. Thermal sensation was measured using a 9‐point scale, where 4 = very hot, 3 = hot, 2 = warm, 1 = slightly warm, 0 = neutral, −1 = slightly cool, −2 = cool, −3 = cold, and − 4 = very cold. Additional measurements were taken and are included in Figures S1 and S2 for context, including thermal discomfort, mean skin temperature, and local skin temperatures for the tricep, abdomen, chest, subscapula, cheek, forehead, thigh, calf, foot, and big toe. Thermal discomfort was measured with a 5‐point scale, where 0 = comfortable, 1 = slightly uncomfortable, 2 = uncomfortable, 3 = very uncomfortable, and 4 = intolerable. A 13‐site mean skin temperature was calculated per Equation 1 (Castellani et al., 2018).

(1) $$\begin{array}{cc} \text{Mean skin temperature}= & 0.0215\;\left(\text{Finger}\right)+0.0285\;\left(\text{Hand}\right)+0.07\;\left(\text{Forearm}\right)+0.07\;\left(\text{Tricep}\right)\\ & +0.116\;\left(\text{Abdomen}\right)+0.117\;\left(\text{Chest}\right)+0.117\;\left(\text{Subscapula}\right)+0.035\;\left(\text{Cheek}\right)\\ & +0.035\;\left(\text{Forehead}\right)+0.19\;\left(\text{Thigh}\right)+0.13\;\left(\text{Calf}\right)+0.058\;\left(\text{Foot}\right)+0.012\;\left(\mathrm{Toe}\right) \end{array}$$

### 2.4 Experimental design and procedures

**FIGURE 1:**
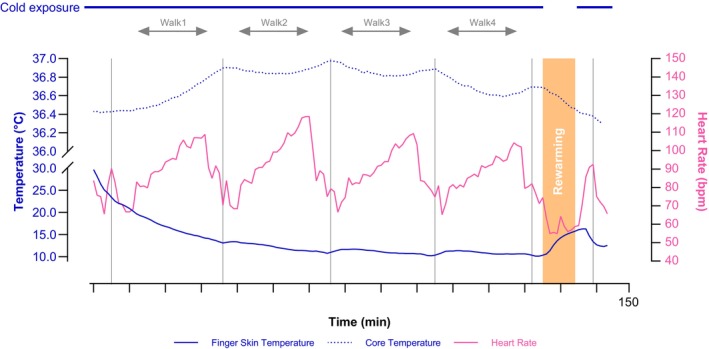
Representative example of one participant in a 0°C trial. Individuals participated in three experimental trials that differed only by air temperature (20°C, 10°C, and 0°C). Participants (3 females and 11 males) engaged in ~130 min of physical activity where dexterity was assessed at five timepoints (DT1–5) throughout the exposure, followed by a 10‐min seated passive rewarming period (~22°C air temperature), and ending with a final dexterity assessment (DT6) upon re‐entry to the cold environment. Physical activity consisted of 4 cycles of a ~10 min performance battery with 20 min treadmill walking at varying speeds and grades [walk 1–4], after which a final performance battery was performed. The performance batteries consisted of 3 sets of 1 repetition (3 × 1) of squat jumps (30‐s rest between repetitions), 3 × 1 isometric mid‐thigh pull (30‐s rest between repetitions), seated dexterity test, and 1 × 3 maximal isometric handgrip strength on each hand (15‐s rest between repetitions). Representative finger skin temperature, core temperature, and heart rate data from one participant's 0°C trial are shown to highlight the modest increases in core temperature and reductions in finger skin temperature that occurred with varying exercise intensities throughout the trial.

The present study was part of a larger ongoing protocol (Chapman, Schafer, et al., 2024; Looney et al., 2024, 2025; Schafer et al., 2025), with brief descriptions of the protocol that are outside the aim of the present study included for context. Figure 1 graphically shows the experimental overview. The present study employed a randomized, crossover design where individuals participated in one familiarization visit, one baseline visit, and three experimental visits where the environmental conditions were randomized. All visits were separated by ≥48 h to ensure sufficient recovery and mitigate any potential influence from previous visits. All trials commenced between 0500 and 0700 h and at the same morning time for a given participant to control for the time of day. Additionally, participants were required to refrain from (1) high‐intensity exercise for >48 h; (2) alcohol for >24 h; and (3) caffeine, nicotine, and food intake for ≥10 h. Euhydration upon arrival to a study visit was encouraged by instructing participants to consume ≥500 mL of water both the night before and the morning of each visit. Participants provided a urine sample upon arrival to all visits to ensure urine specific gravity was ≤1.030. This cutoff was selected to increase the generalizability of our findings given the high prevalence of mild dehydration in free‐living adults (Chapman et al., 2023).

The objective of this study was to quantify the physiological and performance responses to progressive cold strain during prolonged light‐to‐moderate intensity physical work in cold environments using laboratory‐controlled conditions. The minimal operating air temperature of the climatic chamber was 0°C. Thus, to ensure sufficient cold strain was induced during this experimental paradigm, participants wore combat boots and standard light physical training attire (i.e., shorts, t‐shirt, and socks or the Army Physical Fitness Uniform) (thermal values: 0.91 clo; 0.51 i_m_/clo) (Potter et al., 2016). Participants also wore lightweight (88% polyester and 12% spandex), commercially available, running gloves (B07WNM4FXW, CEVAPRO, Amazon, Seattle, WA) on their hands to improve comfort over the duration of the study. To establish an optimal response scenario, the interactive relationships between the environment and clothing selected were modeled to support the protocol development (Potter et al., 2020; Xu et al., 2021). Specifically, the modeling conducted in the study design phase predicted that the clothing used in the present study would elicit similar skin temperature responses during a minimally dressed exposure to 0°C compared to an appropriately clothed exposure to −20°C. Participants kept the gloves on at all times during the experimental visits, including during dexterity testing and rewarming periods.

The procedures for the familiarization (visit 1) and baseline (visit 2) visits were the same. Basal metabolic rates were measured for 30 min as previously described (Chapman, Schafer, et al., 2024; Looney et al., 2025). Then, participants entered the climate‐controlled chamber (20.3 ± 0.7°C and 49 ± 5% relative humidity) and performed one round of a battery of physical performance tasks, followed by 20 min of graded treadmill walking, and then one final round of the performance battery. These activities simulated occupational work demands by testing an array of maximal strength and dexterity performance indicators and light‐to‐moderate physical activity. The performance battery included the following workflow: (1) three static squat jumps with 30 s rest between each repetition; (2) three maximal effort isometric mid‐thigh pulls for ~3–4 s with 30 s rest between each repetition; (3) manual dexterity assessment via one round of the Placing Test from the Complete Minnesota Manual Dexterity Test (Model 32023A, Lafayette Instrument, Lafayette, IN); (4) three repetitions on each hand of standing maximal effort isometric handgrip strength testing with 15 s between each repetition; and (5) perceptual measures (e.g., thermal sensation). The dexterity test was conducted according to the manufacturer's instructions, which the exception of performing the test once per time period for logistical reasons. In brief, the Placing Test is a reliable measure (Fleishman & Ellison, 1962) of manual dexterity that requires participants to be in a seated position at a table to move 60 plastic disks one at a time from one testing board to another using only their dominant hand. The Placing Test is a validated assessment of manual dexterity, which involves the coordination of the entire hand, including fingers, thumb, and wrist, and related skills (e.g., grip strength, hand‐eye coordination, and the ability to perform tasks involving the entire hand). The performance battery was immediately followed by 20 min of treadmill walking that included five 4‐min stages at ascending vertical speeds of 0.00, 1.93, 3.86, 5.79, and 7.79 m∙min^−1^ that were randomized as described elsewhere (Looney et al., 2025). Upon finishing the treadmill walk, participants completed the performance battery one more time following the same previously described procedures.

The three experimental visits (visits 3–5) differed only by the environmental temperature (i.e., 20°C, 10°C, and 0°C) and were randomized and counterbalanced between participants. After providing written confirmation of adherence to the protocol restrictions, participants were instrumented with physiological monitors to continuously measure core temperature, skin temperature, and heart rate during the protocol. Then, participants entered the climatic chamber. Participants were allowed to drink water ad libitum throughout the experiment. The measured conditions of the climatic chamber for each trial were 20°C: 20.3 ± 0.3°C and 50 ± 4% relative humidity, 10°C: 9.8 ± 0.2°C and 56 ± 5% relative humidity, 0°C: 1.3 ± 1.4°C and 40 ± 7% relative humidity with air circulating at ~1.3 m⋅s^−1^. Upon entering the chamber, participants engaged in four rounds of performing one performance battery (~10 min) and one 20 min graded treadmill walk using the same sequence described for the familiarization and baseline testing visits. After the fourth treadmill walk, a fifth performance battery was performed. Then, participants exited the climatic chamber and sat in a chair for 10 min of spontaneous rewarming in the antechamber with environmental conditions of 20°C: 23.1 ± 0.7°C and 41 ± 9% relative humidity, 10°C: 21.7 ± 0.9°C and 37 ± 8% relative humidity, 0°C: 20.9 ± 1.4°C and 35 ± 12% relative humidity. Thermal sensation was recorded at minute 9 of rewarming. After 10 min of spontaneous rewarming, participants re‐entered the environmental chamber and engaged in one final performance battery.

### 2.5 Data analysis

Core temperature, skin temperature, and heart rate data were converted to minute‐by‐minute averages. To reduce the effect of interindividual variability on the dexterity test, we assessed dexterity as the absolute and percent changes from baseline during each of the six performance batteries (i.e., the five batteries before rewarming and the final battery after rewarming). Baseline dexterity was quantified as the time to completion for the final dexterity test on visit 2, which represented a given participant's best performance for the dexterity test as it was performed in ideal conditions and it limited the potential for a learning effect to influence the results (i.e., this was the fourth time that participants performed the test). Moreover, given that skin temperature is rapidly reduced upon entering cold environments, this approach also allowed for the inclusion of the change from baseline data from the first battery in our models, rather than using the first battery as baseline when skin temperature is already declining. Given that differences in morphology largely explain sex differences in the thermoeffector responses to cold (Castellani & Young, 2016; Greenfield et al., 2023), male and female data were grouped for statistical analysis.

### 2.6 Statistical analysis

Data were analyzed using R (Version 4.4.1; R foundation for Statistical Computing; Vienna, Austria) and are reported as mean ± standard deviation (SD) unless noted otherwise. The present study was considered a secondary outcome of the larger ongoing study (Chapman, Schafer, et al., 2024; Looney et al., 2024, 2025) and thus an a priori power analysis was not performed for the primary aim presented herein.

The procedures to address our primary aim regarding the influence of rewarming on the relation between extremity temperature and dexterity were as follows. Data were grouped according to whether they occurred before (Before Rewarming) or after (After Rewarming) the rewarming period. The potential for a threshold in the relation between extremity temperature and the absolute or percent change in dexterity time was visualized by fitting a locally estimated scatterplot smoothing curve (shown as insets in figures). The influence of extremity temperature on dexterity was then examined by fitting linear mixed effects models with fixed effects of extremity temperature and random effects of participants on intercepts, with core temperature nested within participants using the “lme4” package (Bates et al., 2015). Core temperature was included in the model because of its likely influence on dexterity (Alba et al., 2019) due to its modulatory effect on cold‐induced vasodilation (Flouris et al., 2008). Body heat storage, which can be reliably estimated using partitional calorimetry (Cramer & Jay, 2019) and is an important modulator of manual dexterity during cold stress (Brajkovic et al., 2001; Brajkovic & Ducharme, 2003), was not calculated due to logistical reasons that prevented the measurement of expiratory gases after the rewarming period. Further, thermometry‐based estimates of heat debt were not used because of the reduced accuracy and reliability in cold environments (Vallerand et al., 1992; Wallace et al., 2024).

Hypothesis testing for the existence of a breakpoint within the segmented regression models was performed using Muggeo's Test (Muggeo, 2016). Then, segmented regression models were used to approximate the statistical extremity temperature threshold for exacerbated decrements in dexterity using the “segmented” package (Muggeo, 2008). After identifying the statistical threshold, the potential for a nonlinear relation between extremity temperature and dexterity decrements at the colder extremity temperatures was assessed. The random effect of participants on intercepts was included in mixed‐effects models with or without a quadratic polynomial function using the “nlme” or “lme4” packages (Bates, 2014; Pinheiro, 2011). An iterative process was used to assess the influence of core temperature in the models as a multiplicative effect, additive effect, or as a random effect nested within participants. Model fits were evaluated and selected using the Akaike information criterion (AIC). Coefficients of determination (*R* ^2^) were calculated per the method of Nakagawa & Schielzeth (2013) for mixed‐effects models. In the event that a statistical threshold was not identified, linear and polynomial regression models were evaluated using AIC.

To account for the longitudinal nature of the data (i.e., repeated measures), receiver operating characteristic (ROC) curves were constructed from the predicted probability of a positive condition occurring within a generalized linear mixed effects model (GLMM) (Liu et al., 2022) that included fixed effects of test number (to account for the timing and order of the dexterity test) and random effects of participants on intercepts using the “lme4” and “pROC” packages (Bates et al., 2015; Robin et al., 2011). The GLMM approach avoided the potential for a modest inflation in the diagnostic accuracy analysis to occur when not accounting for repeated measures (Chapman, Johnson, et al., 2024; Liu & Wu, 2003). The area under the ROC curve (AUC, mean with 95% confidence intervals) was used to quantify the ability of a given cold strain indicator to discriminate a 5% or 10% reduction in dexterity. The Placing Test has an estimated coefficient of variation of 4.1% when performed once (Jurgensen, 1943) and thus, in considering signal‐to‐noise of the dexterity test, the 5% reduction in dexterity represents the minimal decrement in dexterity that was deemed to be clinically meaningful and outside of the expected measurement variability. Diagnostic accuracy was interpreted as AUC of 0.5 or less indicates no discrimination, 0.5–0.7 is considered poor, 0.7–0.8 is considered acceptable, 0.8–0.9 is considered excellent, and above 0.9 is considered outstanding (Hosmer Jr. et al., 2013). Furthermore, to consider the potential influence of brief rewarming on the diagnostic accuracy of these measurements, independent ROC curves were constructed with the inclusion of post‐rewarming dexterity tests (i.e., all six dexterity tests were included) or excluding the final dexterity test (i.e., only tests 1–5 used). An independent ROC curve on the post‐rewarming data only was not constructed given that the present study was underpowered for that analysis (i.e., only one dexterity test was performed after rewarming), because that was considered outside the scope of the larger protocol when designing the study.

In a few instances, technical difficulties impeded syncing the core temperature telemetry pill data with the device receiver, resulting in some missing 15‐s intervals of data. These data were imputed using linear interpolation to avoid overfitting. Additionally, technical difficulties resulted in missing data for core temperature in *n* = 1 trial (0°C) and finger skin temperature in *n* = 1 trial (10°C). These data were not imputed. The α‐level for statistical significance was set to 0.05.

## 3 RESULTS

**FIGURE 2:**
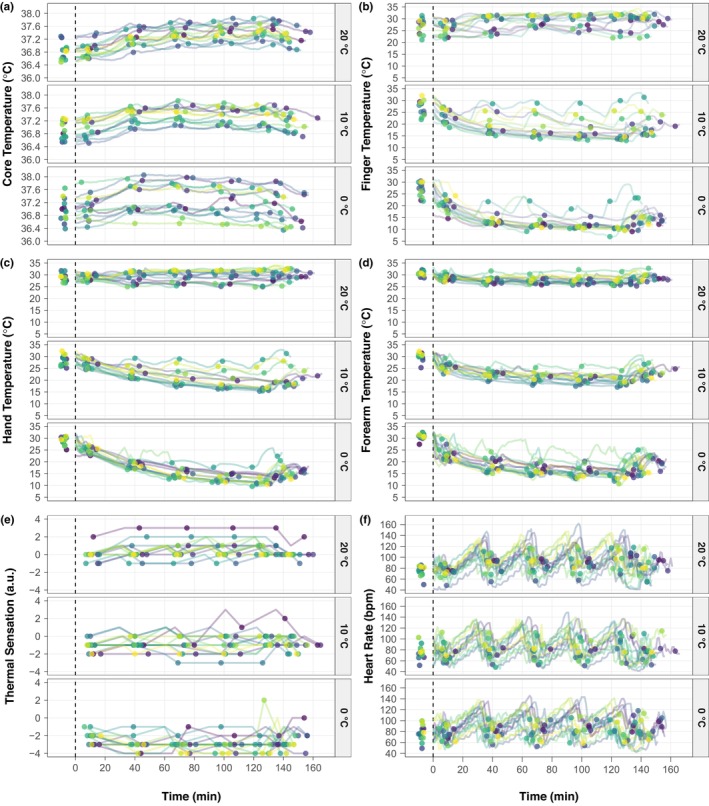
Descriptive individual physiological responses to occupational cold stress and rewarming. Participants engaged in ~130 min of physical activity in the cold in which dexterity was assessed at five timepoints throughout the exposure, followed by a 10‐min passive rewarming period, and ending with a final dexterity assessment upon re‐entry to the cold environment. Individuals participated in three experimental trials that differed only by air temperature 20°C, 10°C, and 0°C). The dashed lined separates baseline values prior to insertion in the cold environment (on the left) and values across time during the prolonged cold exposure, rewarming period, and subsequent reinsertion into the cold environment (on the right). Individual data are presented by lines. Circles on the right of the dashed line depict when the dexterity tests occurred for a given participant (six total per a given environmental condition). Note that thermal sensation was not measured prior to insertion into the cold environment. *n* = 14 (3 females and 11 males).

The descriptive individual responses of measures of cold strain most relevant to the present study are shown in Figure 2. Before rewarming, the increases in core temperature to cold exposure was relatively similar across the three environmental conditions, whereas after‐drop (i.e., post‐cold exposure reductions in core temperature) was visually observed in the 0°C trial following the rewarming period (Figure 2a). Local skin temperature exhibited a dose–response relation whereby the fingers, hands, and forearm reached progressively lower temperatures (Figure 2b–d) and felt colder (Figure 2e) from the 20°C to 0°C conditions. The heart rate responses show that participants experienced a range of exercise intensities throughout the experiments (Figure 2f). Additional local skin temperature, 13‐site weighted mean skin temperature, and thermal discomfort data can be found in Figures S1 and S2.

**FIGURE 3:**
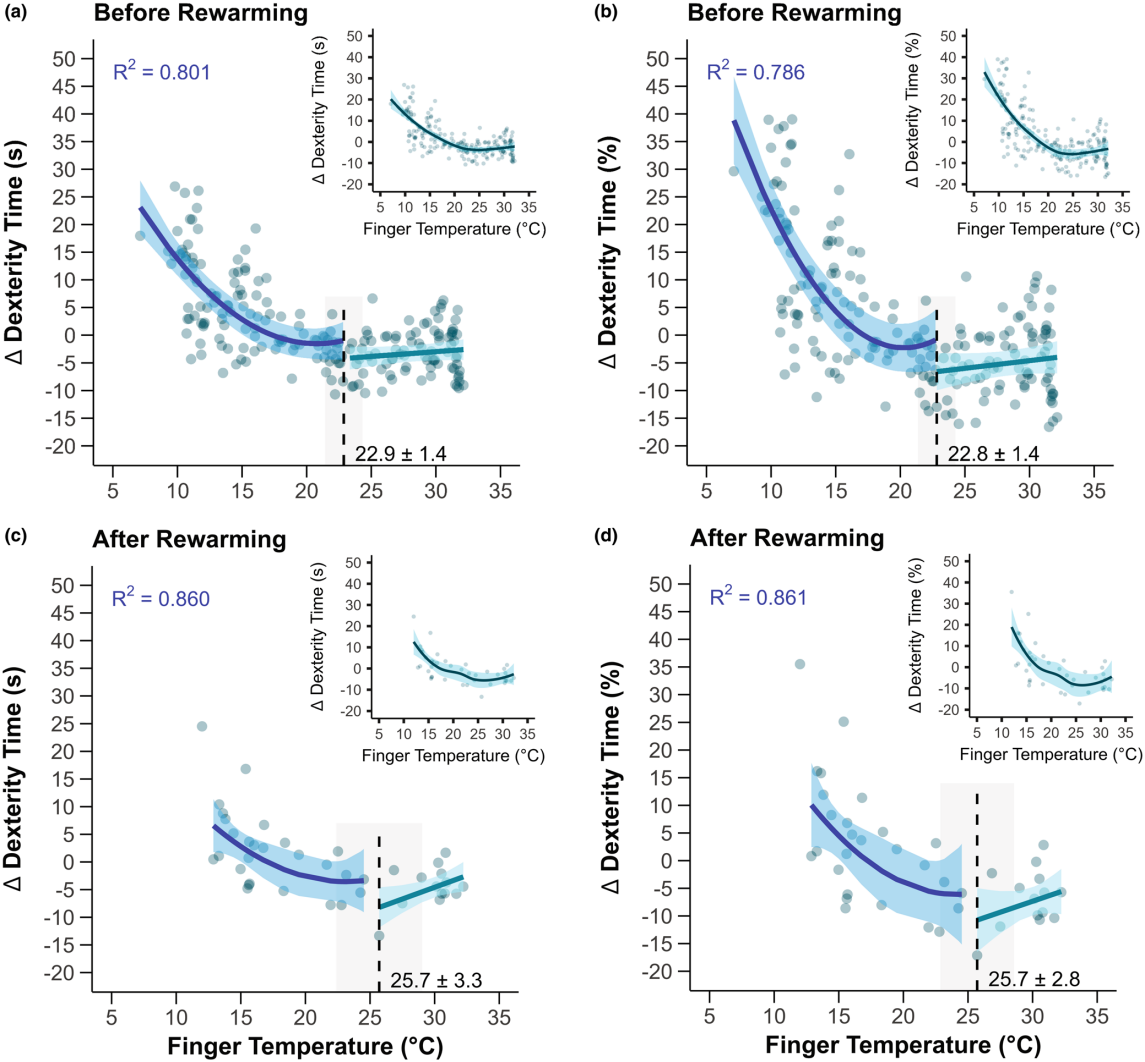
Finger skin temperature thresholds for greater losses of dexterity during occupational cold stress. Participants engaged in ~130 min of light‐to‐moderate physical activity in the cold in which dexterity was assessed at five timepoints throughout the exposure (Before Rewarming, panels a and b), followed by a 10‐min passive rewarming period, and ending with a final dexterity assessment upon re‐entry to the cold environment (After Rewarming, panels c and d). The change (∆) in the time to complete the dexterity test compared to a given participant's best performance during a baseline visit is expressed in seconds (Panels a and c) or percent change (Panels b and d). Main panels display the statistical threshold point estimate with standard error (dashed line with gray shading and values presented), with model fit from segmented regression in green (mean with 95% confidence intervals) and best model fit after the threshold by polynomial regression (*R* ^2^ values presented) in blue (mean with 95% confidence intervals). Inset figures show the same data for a given panel with the application of a locally estimated scatterplot smoothing curve to aid visualization. *n* = 14 (3 females and 11 males).

During progressive cold strain without rewarming, the statistical threshold for when a greater loss of dexterity occurred for a given reduction in skin temperature was ~22.9°C for finger temperature (Figure 3a,b, *p* < 0.0001). Following rewarming, the threshold for finger temperature and dexterity loss was ~25.7°C (Figure 3c,d, *p* ≤ 0.007). In both conditions, polynomial regression after the statistical threshold improved the model fit compared to the segmented linear regression model, with *R* ^2^ values from the curvilinear fit after the identified threshold reported in Figure 3.

**FIGURE 4:**
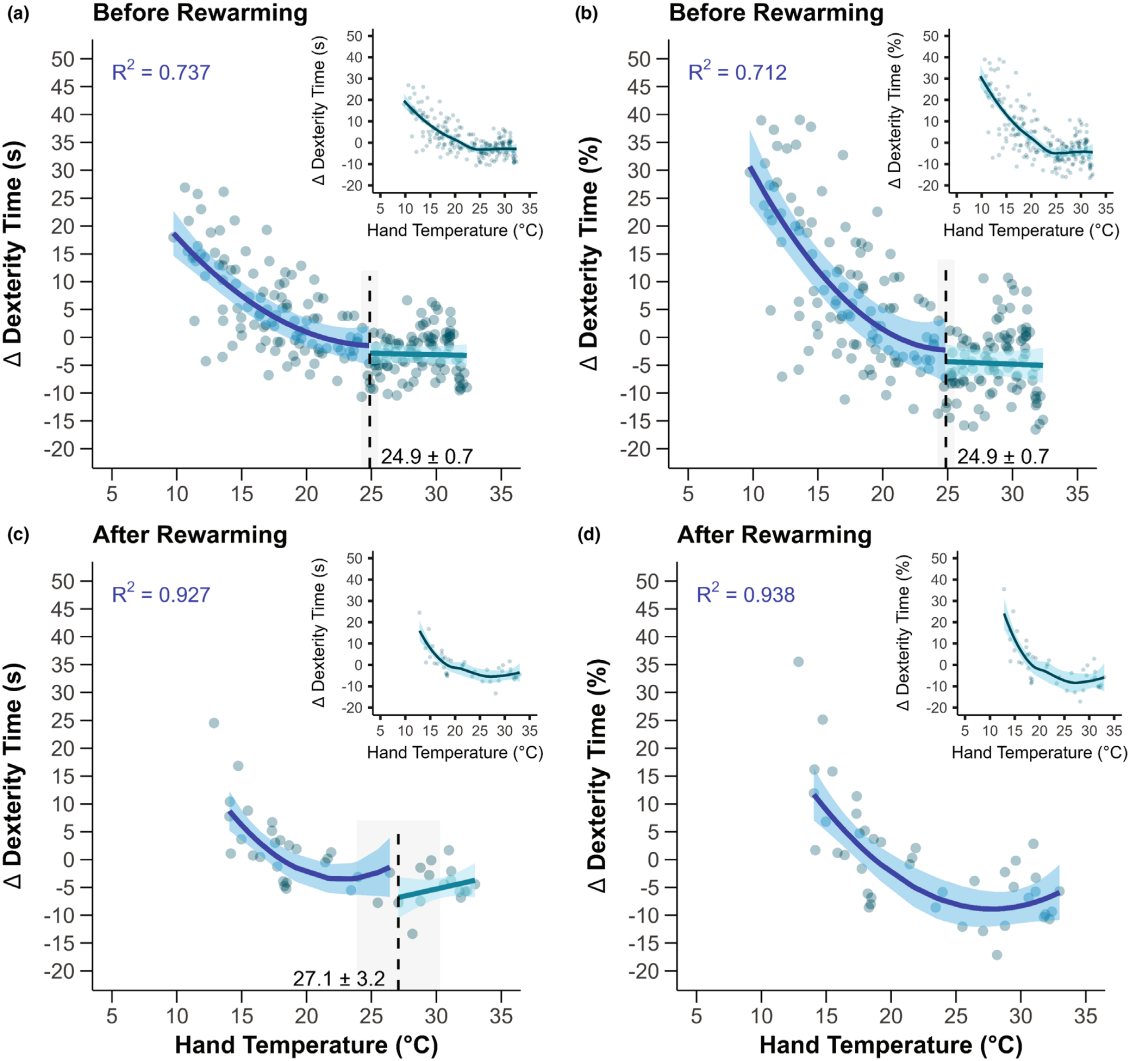
Hand skin temperature thresholds for greater losses of dexterity during occupational cold stress. Participants engaged in ~130 min of light‐to‐moderate physical activity in the cold in which dexterity was assessed at five timepoints throughout the exposure (Before Rewarming, panels a and b), followed by a 10‐min passive rewarming period, and ending with a final dexterity assessment upon re‐entry to the cold environment (After Rewarming, panels c and d). The change (∆) in the time to complete the dexterity test compared to a given participant's best performance during a baseline visit is expressed in seconds (Panels a and c) or percent change (Panels b and d). Main panels display the statistical threshold point estimate with standard error (dashed line with gray shading and values presented), with model fit from segmented regression in green (mean with 95% confidence intervals) and best model fit after the threshold by polynomial regression (*R* ^2^ values presented) in blue (mean with 95% confidence intervals). Inset figures show the same data for a given panel with the application of a locally estimated scatterplot smoothing curve to aid visualization. *n* = 14 (3 females and 11 males).

In the hands, progressive cold strain without rewarming elicited a threshold of ~24.9°C for hand skin temperature for greater reductions in dexterity (Figure 4a,b, *p* < 0.0001). After rewarming, a statistical threshold for hand skin temperature was identified at ~27.1°C when data were expressed in seconds (Figure 4c, *p <* 0.001), but a threshold was not identified when data were expressed as percent change (Figure 4d, *p* = 0.074). Similar to the fingers, the model fit after the hand skin temperature threshold was improved by polynomial regression, with *R* ^2^ values of this curvilinear fit reported in Figure 4a,b. Following rewarming, polynomial regression also improved model fit for data expressed in seconds with threshold (Figure 4c) or as percent change where a threshold was not identified (Figure 4d).

**FIGURE 5:**
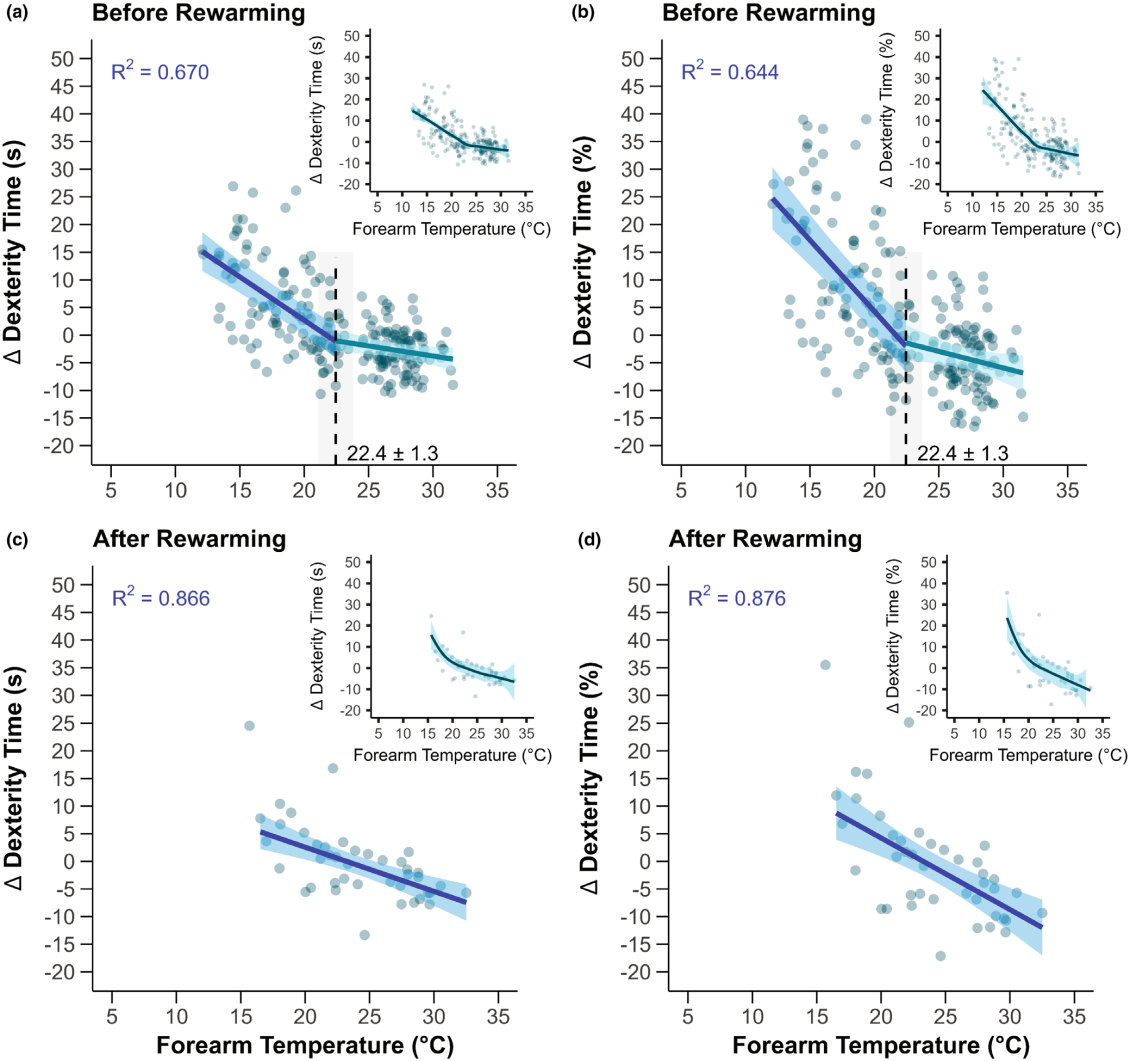
Forearm skin temperature thresholds for greater losses of dexterity during occupational cold stress. Participants engaged in ~130 min of light‐to‐moderate physical activity in the cold in which dexterity was assessed at five timepoints throughout the exposure (Before Rewarming, panels a and b), followed by a 10‐min passive rewarming period, and ending with a final dexterity assessment upon re‐entry to the cold environment (After Rewarming, panels c and d). The change (∆) in the time to complete the dexterity test compared to a given participant's best performance during a baseline visit is expressed in seconds (Panels a and c) or percent change (Panels b and d). Main panels display the statistical threshold point estimate with standard error (dashed line with gray shading and values presented), with model fit from segmented regression in green (mean with 95% confidence intervals) and best model fit after the threshold by polynomial regression (*R* ^2^ values presented) in blue (mean with 95% confidence intervals). Inset figures show the same data for a given panel with the application of a locally estimated scatterplot smoothing curve to aid visualization. *n* = 14 (3 females and 11 males).

The forearm skin temperature threshold for greater dexterity loss during progressive cold strain without rewarming was ~22.4°C (Figure 5a,b, *p* ≤ 0.002). However, unlike the fingers and hands, a threshold was not identified following forearm skin temperature rewarming (Figure 5c,d, *p* ≥ 0.058). Additionally, before rewarming, the best model fit for forearm skin temperature was the segmented regression without a curvilinear component (Figure 5c,d).

**FIGURE 6:**
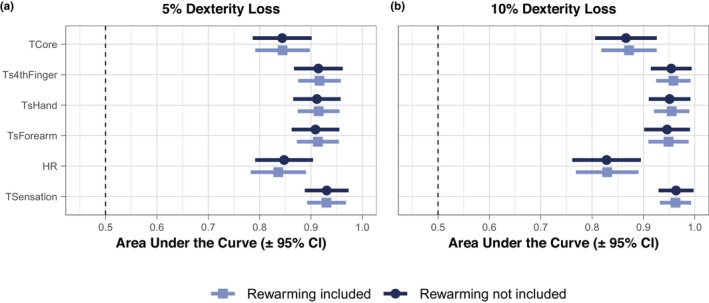
Diagnostic accuracy of skin temperature, core temperature, and thermal sensation for discriminating dexterity loss during cold exposure and rewarming. Participants engaged in ~130 min of physical activity in the cold in which dexterity was assessed at five timepoints throughout the exposure, followed by a 10‐min passive rewarming period, and ending with a final dexterity assessment upon re‐entry to the cold environment. The ability of cold strain measurements to discriminate a 5% (left panel) or 10% (right panel) loss of dexterity is plotted as the area under the curve with 95% confidence intervals from receiver operating characteristic curves constructed from a generalized linear mixed effects model to account for repeated measures. Diagnostic accuracy for dexterity was assessed with inclusion of the rewarming data (i.e., all six dexterity tests) or exclusion of the rewarming data (i.e., dexterity tests 1–5). TCore, core temperature; Ts4thFinger, 4th finger skin temperature; TSensation, thermal sensation; TsHand, hand skin temperature. *n* = 14 (3 females and 11 males).

The diagnostic accuracy of cold strain to discriminate a 5% or 10% loss of dexterity is presented in Figure 6. The diagnostic accuracy of all cold strain measurements was not altered by the inclusion or exclusion of rewarming data in the analysis (Figure 6). Skin temperature exhibited outstanding diagnostic accuracy (AUC ≥ 0.90) across all sites for discriminating a 5% loss in dexterity (Figure 6). The already outstanding diagnostic accuracy of skin temperature was slightly improved numerically for discriminating a 10% loss in dexterity compared to 5% (Figure 6). Core temperature and thermal sensation exhibited outstanding diagnostic accuracy at both thresholds of dexterity loss (Figure 6). The diagnostic accuracy of the additional local skin temperatures, 13‐site weighted mean skin temperature, and thermal discomfort data can be found in Figure S3.

## 4 DISCUSSION

The objective of this study was to identify extremity skin temperature thresholds where dexterity loss precipitously occurs during non‐hypothermic occupational cold stress and rewarming, and to determine the diagnostic accuracy of cold strain measurements for discriminating a 5% and 10% loss of dexterity. Several novel findings were observed. First, the skin temperature thresholds whereby greater losses in dexterity occur were finger temperatures of ~22.9°C, hand temperatures of ~24.9°C, and forearm temperatures of ~22.4°C. Second, finger and hand temperatures had a curvilinear relation to dexterity loss at these thresholds, whereas forearm temperature had a linear relation. Third, following rewarming, the present findings suggest that dexterity decrements begin at higher skin temperature thresholds in the fingers (~25.7°C), but these thresholds in the hand and forearm appear to be eliminated following rewarming. Further research is needed to confirm the findings of the rewarming period with a larger sample given that the study design allowed for only one testing period upon reinsertion to the cold environment. Fourth, we found that the outstanding diagnostic accuracy of extremity skin temperature in discriminating clinically meaningful reductions in dexterity (i.e., 5% and 10%) was not affected by a brief passive rewarming, which supports the use of skin temperature to estimate dexterity changes during cold work–warm rest cycles during occupational cold stress.

The present study identified that, in non‐hypothermic conditions, finger temperatures of ~22.9°C, hand temperatures of ~24.9°C, and forearm temperatures of ~22.4°C as the thresholds for greater dexterity loss. These values are higher than the typically reported finger temperature thresholds between 12°C and 20°C (Ray et al., 2019), although 15°C is typically regarded as the finger temperature threshold for marked decrements in dexterity (Castellani & Tipton, 2016; TBMED‐508, 2005), and hand temperatures around 22°C (Kingma et al., 2023). The higher thresholds reported in the present study are likely due to a number of factors, including differences in the cold exposure paradigm (e.g., resting vs. exercise, water immersion vs. cold air), statistical (or visual in some studies) approaches used for identifying thresholds, the location of skin temperature measurements, and the test administered to assess dexterity. The present study used an objective statistical approach to identify the presence of a threshold and then compared best fitting models at the lower skin temperatures during a prolonged non‐hypothermic exposure involving light‐to‐moderate physical activity in a cold environment. The thresholds for finger temperatures are likely dependent on the location of the finger temperature, given that the present study used the dorsal aspect of the proximal phalanx of the fourth finger, whereas other studies may have used the fingertip or an unspecified anatomical finger location (Castellani & Tipton, 2016). Nonetheless, temperature thresholds identified in the present study align with a recent study that demonstrated that maximal cutaneous vasoconstriction is observed at local skin temperatures between ~23°C and 27°C (Massey et al., 2018). During cold stress, reductions in skin temperature cause a rapid reflex cutaneous vasoconstriction to mitigate heat loss prior to the activation of cold‐induced thermogenesis or behavioral thermoregulation (Schlader et al., 2016). Within 5–10 min of cold exposure, cyclic vasodilation predominantly in the fingers (i.e., cold‐induced vasodilation [CIVD]) becomes an important physiological response that is modulated by body surface area‐to‐mass ratio (Weller et al., 2024) and body heat content (Flouris et al., 2008) to buffer reductions in finger skin temperatures. We were unable to measure CIVD in the present study for logistical reasons. Given that finger blood flow has an important role in dexterity during cold exposure (Brajkovic & Ducharme, 2003), further research is warranted to tease apart the influence of CIVD on dexterity recovery during cold work–warm rest cycles experienced during occupational cold stress.

The present study also lends support that finger skin temperature thresholds for greater loss of dexterity are increased following a brief rewarming. Further, the existence of a skin temperature threshold for the hands and forearms also appears to change with rewarming, at least in our experimental model of brief rewarming. Although not measured in the present study, we speculate that the differing skin temperature thresholds following rewarming are mediated by dynamic changes in body heat content over this period (Flouris et al., 2008). This hypothesis is supported by the marked changes to skin and core temperatures upon rewarming and upon reinsertion to the cold environment (Figure 1). Given that the present study design only allowed for one dexterity test in each condition, future studies are needed to confirm this hypothesis and confirm in a large sample whether skin temperature thresholds increase or are altered by reinsertion to a cold environment. A major strength of the present investigation was the integration of multimodal exercises of varying intensities and intermittent work performed during prolonged cold air exposure, including a brief passive rewarming period followed by reentry to the cold environment that together simulated the anticipated demands of occupational cold stress. This approach allowed for the quantification of skin temperature and other cold strain measurements across a range of temperatures (or values) and time under exposure to better tease apart the thresholds at which dexterity loss precipitously occurs during occupational cold stress and rewarming. It has been well reported that reductions in skin temperature are associated with large reductions in manual dexterity across a range of exposures, including cold air or cold water (Castellani & Tipton, 2016; Cheung, 2015; Havenith et al., 1995; Sullivan‐Kwantes et al., 2021), largely due to local tissue temperatures versus moderate reductions in core temperature (O'Brien et al., 2007). The present study provides novel evidence for extremity skin temperature thresholds during prolonged physical work that is intermittent and varying in intensity during cold exposure and rewarming, as may commonly be experienced in occupational, military, athletic, and recreational settings.

To our knowledge, the present study provides a novel finding from the diagnostic accuracy analysis for discriminating a 5%–10% reduction in dexterity through cold strain measurements that may be monitored during prolonged occupational cold stress and rewarming. As previously discussed, the 5% dexterity loss represented the minimal clinically meaningful reduction in dexterity that is outside the expected test–retest variation of the Placing Test. We found that extremity skin temperature (i.e., finger, hand, and forearm) provides outstanding diagnostic accuracy. Further, core temperature and thermal sensation also display excellent diagnostic accuracy for discriminating dexterity loss. A logical next step is to incorporate the predictive ability of local skin temperature for manual dexterity loss into models to estimate the duration of rewarming needed to replete dexterity. For example, investigators at the U.S. Army Research Institute of Environmental Medicine have developed several models for assessing thermoregulatory responses to environmental stress (Six Cylinder Model and Finite Element Thermoregulatory Model [FETM]) (Castellani et al., 2021; Xu & Werner, 1997) and for the prevention and management of cold weather injuries (Cold Weather Ensemble Decision Aid [CoWEDA]) (Xu et al., 2021). The inclusion of predicted manual dexterity loss and rewarming efficacy would improve mission planning for military leaders and could be leveraged in occupational settings to improve workplace productivity and potentially reduce injuries that may otherwise occur with loss of dexterity. In this regard, future work is needed to understand how external heating devices may change these relationships as heat the forearm area (Castellani et al., 2018) or hands (Wang et al., 2024) has shown efficacy in attenuating reductions in both manual dexterity and local skin temperatures during cold exposure. Finally, an additional future direction of this research would be to extend the important work of Daanen (2009), who determined reliable combinations of exposure duration and Wind Chill Equivalent Temperature (WCET) that correspond to precipitous reductions in finger dexterity at estimated finger skin temperatures of 14°C.

There are a few methodological considerations worth discussing. First, the clothing attire in the present study is unlikely to be worn during occupational cold stress, especially during military training or operations. The climatic chamber used in the present study had a minimum operating temperature specification of 0°C and thus, minimal clothing was required to ensure that the environmental conditions elicited sufficient cold strain among the participants. Nonetheless, to improve ecological validity of the dexterity test, participants wore light gloves throughout the exposure to better reflect scenarios where individuals are not degloving or when degloving is not recommended even for relatively quick tasks. Second, we are unable to assess the influence of finger and hand anthropometrics on dexterity in our paradigm (Payne et al., 2018). However, it is less likely that finger dimensions had a large role in the rewarming data, as dexterity during rewarming occurred within 5 min of re‐entry, and recent evidence suggests that finger dimensions are not a primary mediator of the skin temperature and blood flow responses of the fingers during this early phase of rewarming in warm air following cold water immersion of the hands (Wickham & Cheung, 2023). Third, the present study design allowed for only one dexterity test to be performed during the initial re‐entry to the cold environment following rewarming, resulting in fewer datapoints compared to our before rewarming data. Additional research is needed to confirm the rewarming findings of the present study as well as during longer cold exposures after rewarming. Optimizing rewarming in these settings is important because slower rewarming speeds of the fingers is associated with a higher incidence of cold injury (Brändström et al., 2008). Whether these findings translate to other areas of the body requires future study, as, for example, there is contrasting evidence in the literature regarding the association between hand and foot temperature responses during cold stress (Norrbrand et al., 2017; Van der Struijs et al., 2008). Fourth, we are limited by the relatively small number of females (*n* = 3) who participated in the present study. Although our proportion of participating females is likely low when compared to various occupations exposed to cold environments, that approximately 21% of our sample size was comprised of females is higher than the proportion of females (17.5%) who represent the US Department of Defense active‐duty force (Defense Health Agency, 2022). Fifth, the present study was a laboratory‐controlled study. As such, although we improved ecological validity by incorporating multimodal exercises of varying intensities, the environmental conditions for a given experimental visit were stable. Occupational cold stress may involve numerous dynamic factors that change throughout an exposure and modulate cold strain, including wind, wet environments (e.g., precipitation, water immersion, and sweat that makes clothing wet), high altitude, and/or solar radiation (Schafer et al., 2024). Sixth, although the present study was able to identify skin temperature thresholds for compromised dexterity, the present study was unable to identify the mechanism. Future studies should investigate potential mechanisms during work–rest scenarios, such as viscosity of synovial fluid or reduced sensitivity, so that effective countermeasures can be developed. Thus, despite the present study eliciting several novel findings, maximizing human health and performance in the cold operational environment persists as an understudied area of research.

## 5 CONCLUSION

During simulated occupational cold stress, the present study found that the skin temperature threshold for precipitous losses in dexterity in non‐hypothermic scenarios were finger temperatures of ~22.9°C, hand temperatures of ~24.9°C, and forearm temperatures of ~22.4°C. After rewarming; however, these dexterity decrements began at higher skin temperature thresholds in the fingers (~25.7°C), whereas the data indicate that the skin temperature thresholds are eliminated following rewarming. The diagnostic accuracy of skin temperature, which generally had outstanding diagnostic accuracy for discriminating 5% and 10% dexterity losses, was not altered by a brief passive rewarming. Notably, these findings support the efficacy of estimating dexterity changes from skin temperature during cold work–warm rest cycles that are experienced during occupational cold stress.

## FUNDING INFORMATION

The research was supported by funding (MO230230) from the US Army Medical Research and Development Command (USAMRDC) Military Operational Medicine Research Program (MOMRP).

## CONFLICT OF INTEREST STATEMENT

The authors have no conflicts of interest to declare.

## DISCLAIMER

The opinions or assertions contained herein are the private views of the authors and are not to be construed as official or reflecting the views of the Army of the Department of Defense. Any citations of commercial organizations and trade names do not constitute an official Department of the Army endorsement of approval of the products or services of these organizations.

## Supporting information


Figures S1–S3.

## Data Availability

Data are available from the corresponding author upon reasonable request and approval of a data‐sharing agreement.
