# Supplementary material for: Occupational cold stress and rewarming alters skin temperature thresholds for manual dexterity decrements: An exploratory study
Source: Physiol Rep. 2025 May 8;13(9):e70342. doi: 10.14814/phy2.70342 (PMC12059468; doi:10.14814/phy2.70342)
Supplement: Supplementary file 1 — Figures S1–S3. [file PHY2-13-e70342-s001.zip › Supplementary Figures S1-S3.docx]

**Supplementary Figure** **S1.** *Descriptive individual upper body skin temperature responses to occupational cold stress and rewarming.* Participants engaged in ~150 min of physical activity in the cold in which dexterity was assessed at five timepoints throughout the exposure, followed by a 10-min passive rewarming period, and ending with a final dexterity assessment upon re-entry to the cold environment. Individuals participated in three experimental trials that differed only by air temperature 20, 10, and 0°C). The dashed lined separates baseline values prior to insertion in the cold environment (on the left) and values across time during the prolonged cold exposure, rewarming period, and subsequent reinsertion into the cold environment (on the right). Individual data are presented by lines. Circles on the right of the dashed line depict when the dexterity tests occurred for a given participant (six total per a given environmental condition). *n* = 14 (3 females and 11 males).

**Supplementary Figure** **S2.** *Descriptive individual lower body skin temperature, mean skin temperature, and thermal discomfort responses to occupational cold stress and rewarming.* Participants engaged in ~150 min of physical activity in the cold in which dexterity was assessed at five timepoints throughout the exposure, followed by a 10-min passive rewarming period, and ending with a final dexterity assessment upon re-entry to the cold environment. Individuals participated in three experimental trials that differed only by air temperature 20, 10, and 0°C). The dashed lined separates baseline values prior to insertion in the cold environment (on the left) and values across time during the prolonged cold exposure, rewarming period, and subsequent reinsertion into the cold environment (on the right). Individual data are presented by lines. Circles on the right of the dashed line depict when the dexterity tests occurred for a given participant (six total per a given environmental condition). Note that thermal discomfort was not measured prior to insertion into the cold environment. *n* = 14 (3 females and 11 males).

**Supplementary Figure** **S3.** *Diagnostic accuracy of local skin temperature, 13-site weighted mean skin temperature, and thermal discomfort for discriminating dexterity loss during cold exposure and rewarming.* Participants engaged in ~130 min of physical activity in the cold in which dexterity was assessed at five timepoints throughout the exposure, followed by a 10-min passive rewarming period, and ending with a final dexterity assessment upon re-entry to the cold environment. The ability of cold strain measurements to discriminate a 5% (left panel) or 10% (right panel) loss of dexterity is plotted as the area under the curve with 95% confidence intervals from receiver operating characteristic curves constructed from a generalized linear mixed effects model to account for repeated measures. Diagnostic accuracy for dexterity was assessed with inclusion of the rewarming data (i.e., all six dexterity tests) or exclusion of the rewarming data (i.e., dexterity tests 1–5). TsTricep: tricep skin temperature; TsAbdomen: abdomen skin temperature; TsChest: chest skin temperature; TsSubscapula: subscapular skin temperature; TsCheek: cheek skin temperature; TsForehead: forehead skin temperature; TsThigh: thigh skin temperature; TsCalf: calf skin temperature; TsFoot: foot skin temperature; TsBigToe: big toe skin temperature; TsMean: 13-site weighted mean skin temperature; TDiscomfort: thermal discomfort. *n* = 14 (3 females and 11 males).
