# Supplementary figures and images for: Occupational cold stress and rewarming alters skin temperature thresholds for manual dexterity decrements: An exploratory study
Source: Physiol Rep. 2025 May 8;13(9):e70342. doi: 10.14814/phy2.70342 (PMC12059468; doi:10.14814/phy2.70342)

Supplementary Figure S1

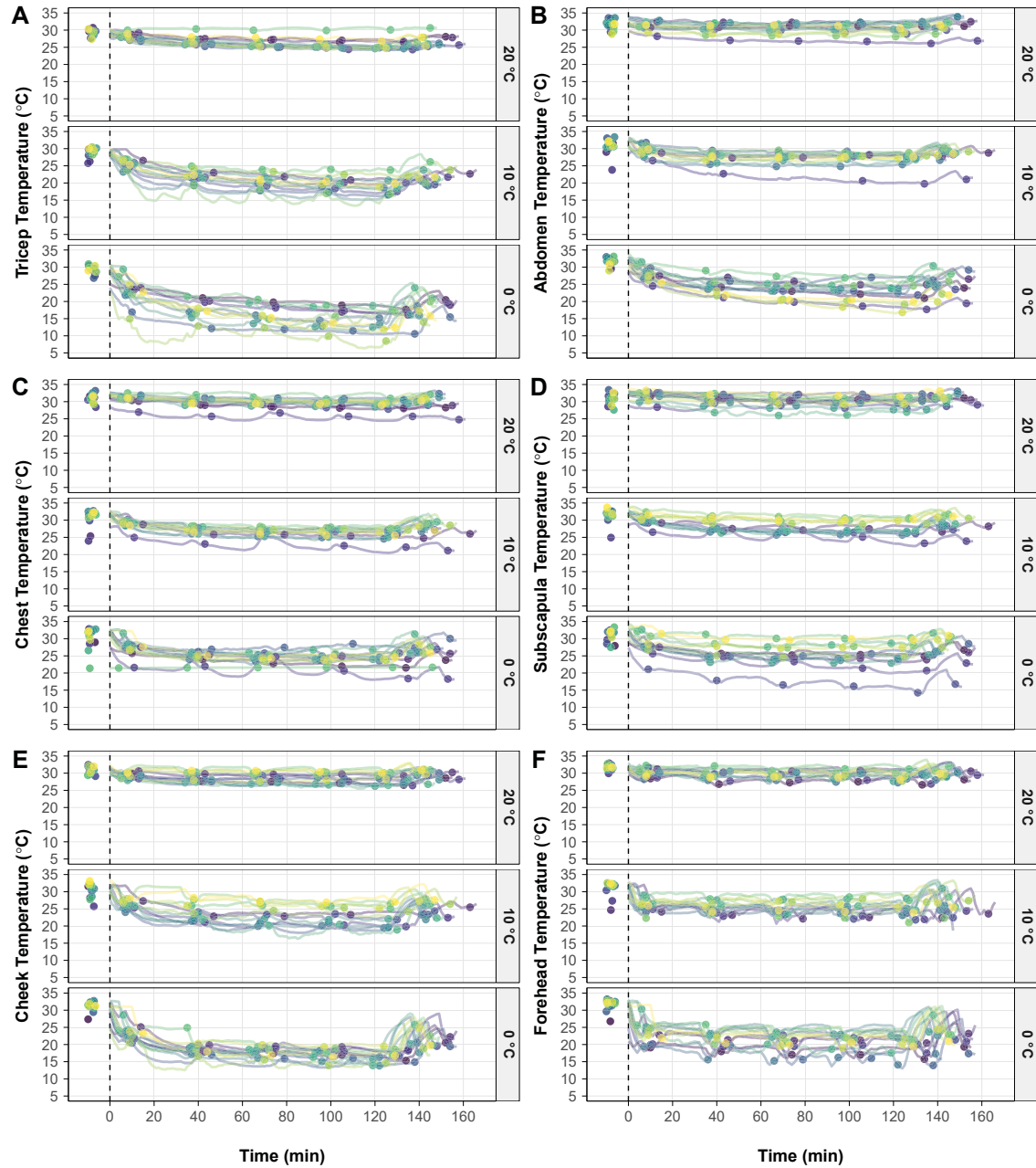

Supplement: Supplementary file 1 — Figures S1–S3. [file PHY2-13-e70342-s001.zip › PHYSREP-2025-03-285-T-f07-z-.pdf]

Supplementary Figure S2

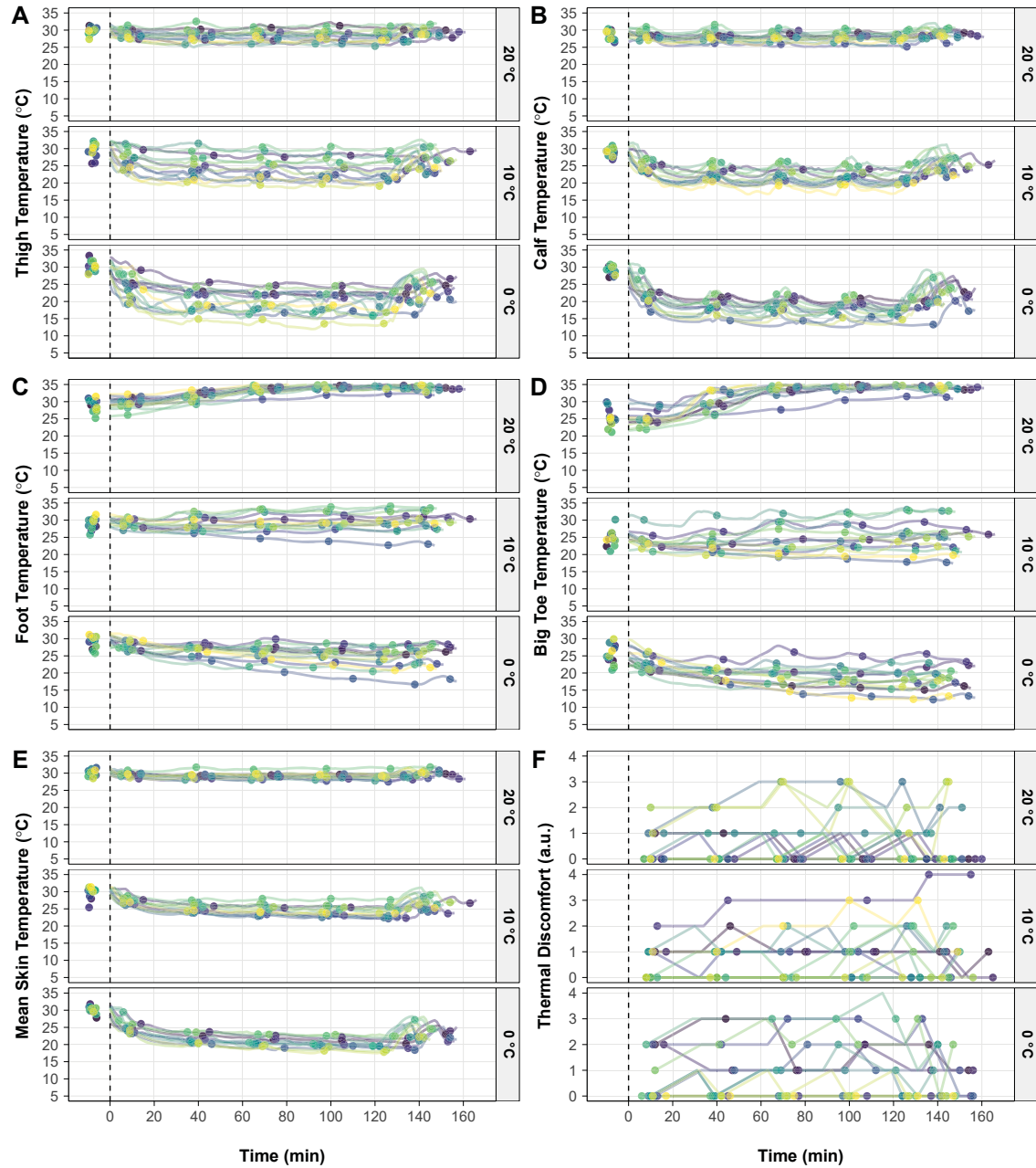

Supplement: Supplementary file 1 — Figures S1–S3. [file PHY2-13-e70342-s001.zip › PHYSREP-2025-03-285-T-f08-z-.pdf]

Supplementary Figure S3

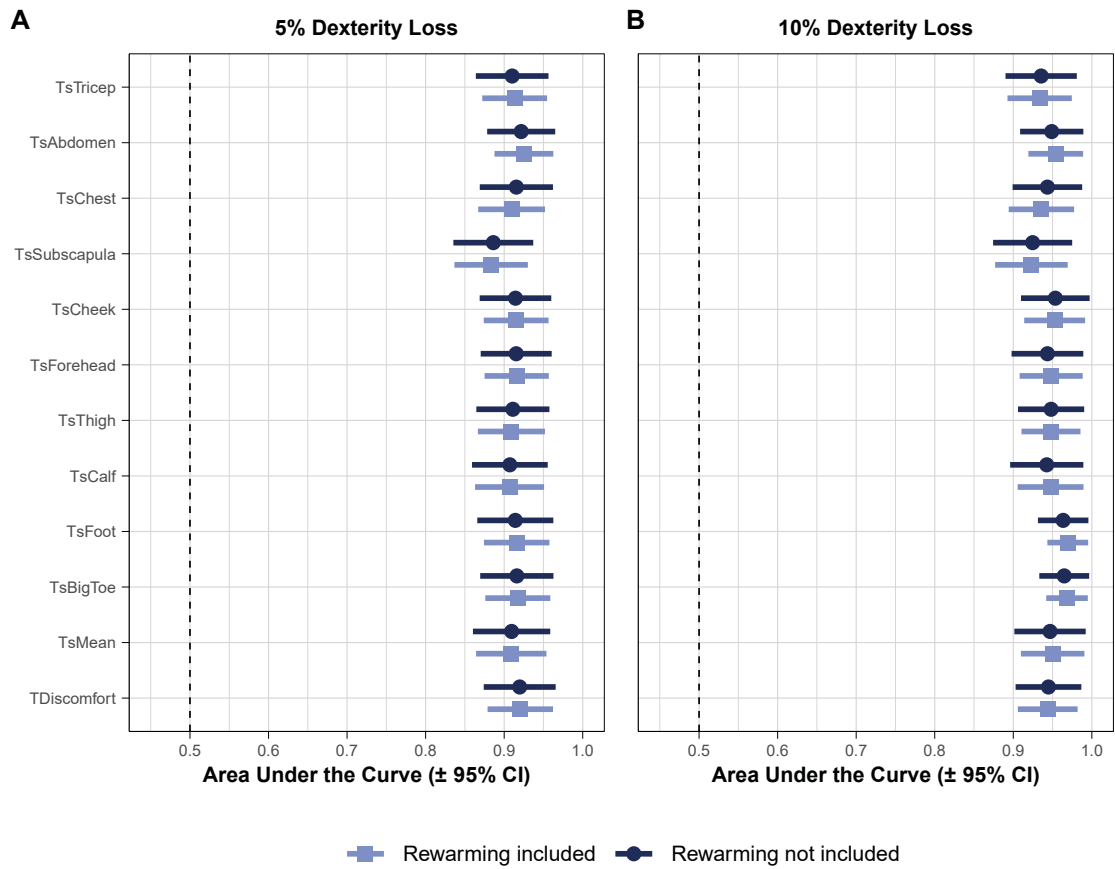

Supplement: Supplementary file 1 — Figures S1–S3. [file PHY2-13-e70342-s001.zip › PHYSREP-2025-03-285-T-f09-z-.pdf]
